# A Cost-Effective ELP-Intein Coupling System for Recombinant Protein Purification from Plant Production Platform

**DOI:** 10.1371/journal.pone.0024183

**Published:** 2011-08-30

**Authors:** Li Tian, Samuel S. M. Sun

**Affiliations:** 1 School of Life Sciences, Tsinghua University, Beijing, China; 2 Life Science Division, Graduate School at Shenzhen, Tsinghua University, Shenzhen, China; 3 School of Life Sciences, The Chinese University of Hong Kong, Shatin, N.T., Hong Kong, China; University of Crete, Greece

## Abstract

**Background:**

Plant bioreactor offers an efficient and economical system for large-scale production of recombinant proteins. However, high cost and difficulty in scaling-up of downstream purification of the target protein, particularly the common involvement of affinity chromatography and protease in the purification process, has hampered its industrial scale application, therefore a cost-effective and easily scale-up purification method is highly desirable for further development of plant bioreactor.

**Methodology/Principal Findings:**

To tackle this problem, we investigated the ELP-intein coupling system for purification of recombinant proteins expressed in transgenic plants using a plant lectin (PAL) with anti-tumor bioactivity as example target protein and rice seeds as production platform. Results showed that ELP-intein-PAL (EiP) fusion protein formed novel irregular ER-derived protein bodies in endosperm cells by retention of endogenous prolamins. The fusion protein was partially self-cleaved *in vivo*, but only self-cleaved PAL protein was detected in total seed protein sample and deposited in protein storage vacuoles (PSV). The *in vivo* uncleaved EiP protein was accumulated up to 2–4.2% of the total seed protein. The target PAL protein could be purified by the ELP-intein system efficiently without using complicated instruments and expensive chemicals, and the yield of pure PAL protein by the current method was up to 1.1 mg/g total seed protein.

**Conclusion/Significance:**

This study successfully demonstrated the purification of an example recombinant protein from rice seeds by the ELP-intein system. The whole purification procedure can be easily scaled up for industrial production, providing the first evidence on applying the ELP-intein coupling system to achieve cost-effective purification of recombinant proteins expressed in plant bioreactors and its possible application in industry.

## Introduction

Production of pharmaceutical proteins in plants has been suggested as an attractive bioreactor platform for its low cost, high yield, large-scale production and reduced health risks in comparison to traditional microbial and mammalian bioreactors, and many valuable recombinant therapeutic proteins have been expressed in transgenic plants as proof-of-concept and feasibility demonstration [1]–[4]. However, to further develop plant bioreactors for large-scale industrial production of recombinant proteins, availability of a cost-effective system for downstream purification of target proteins from plant samples, estimated to account for 80% of the production costs [5], has been a persistent challenge.

The common protein purification method used in plant bioreactors is to express target proteins in fusion with affinity tags, such as His tag and StrepII tag [6], [7] for subsequent affinity purification, but it suffers from difficulty and high cost in scaling up of the required affinity chromatography. Several new fusion strategies to avoid chromatography have been studied and developed, such as oil-body targeting through oleosin fusion [8], two-phase purification through hydrophobin fusion [9] and protein body induction through fusion to γ-zein domain [10]. However, because fusion tags may affect the bioactivity of native proteins, they are generally enzymatically removed from the final protein products by an appropriate protease. This additional cleavage step in purification results in higher cost, in addition to the potential risk of non-specific cleavage of the target protein by the added protease. The development of a simple, scalable and cost-effective downstream recombinant protein purification system is thus highly desirable.

Elastin-like polypeptides (ELP) [11], [12] consist of repeating pentapeptides of V-P-G-X-G (X can be any amino acid except proline) which possess an attractive property of temperature-sensitive phase transition: when temperature is increased to its transition temperature (Tt), the soluble ELP will enter its insoluble phase and self-aggregate, which can be easily pelleted by centrifugation; when temperature is reduced below its Tt , the aggregated ELP will resolubilize and return to its soluble phase. Thus ELP is a good choice to replace the affinity chromatography for its advantages of low-cost and easy scale-up in protein purification. Inteins are a naturally occurring class of protein elements which can catalyze protein self-cleavage [13]–[16]. By amino acid substitution, an intein can be regulated to cleave either at its N- or C-terminus in response to pH shift or thiol reagents [17]. The self-cleavage property of inteins can thus be applied to replace proteolytic cleavage and many intein proteins have been identified for application [18]. The coupling of ELP with intein (Ei tag) in fusion with a target protein becomes a highly attractive system for protein purification in plants: after several cycles of ELP phase transition, the fusion protein can be separated from other proteins through temperature shift and centrifugation; intein is then triggered by pH shift or chemical addition to cleave the target protein from the fusion Ei tag; and finally through another phase transition of ELP and centrifugation, the target protein (in supernatant) can be separated from the Ei tag (as pellet). No protease and special protein purification instruments are needed in the whole procedure, thus simplifying and reducing the time and cost in operation. The ELP-intein fusion strategy has been applied in *E. coli* for recombinant protein purification [19], [20], however, in the last twenty years, although application of ELP or intein as fusion tag for recombinant protein expression and purification in transgenic plants has been studied [21]–[26], there was no report on coupling ELP with intein for protein purification in transgenic plants. Here we report the first successful application of the ELP-intein fusion system in plant for downstream purification of a target recombinant protein.

**Figure 1:**
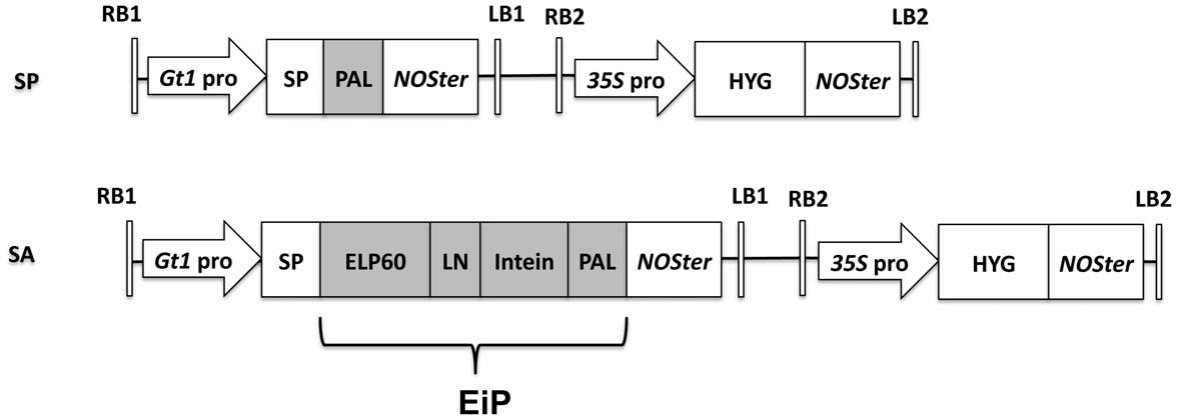
Schematic presentation of expression vectors SP and SA. The expression cassettes for PAL protein, ELP-intein-PAL fusion protein and hygromycin transferase were presented. RB, right border; LB, left border; *Gt1* pro, rice glutelin *Glu*A promoter; *35S* pro, CaMV 35S promoter; LN, linker sequence; HYG, hygromycin phosphotransferase gene; *NOSter*, *NOS* terminator.

In this study, transgenic rice seeds were used as a production system while a lectin protein, designated as PAL (refer to Materials and Methods and protein sequence in Figure S2A) was used as an example target protein. The PAL was expressed in fusion with an Ei tag in the N-terminus, referred to as Ei-PAL or EiP (Figure 1), wherein ELP contained 60 repeating “VPGXG” peptides while intein protein undertook cleavage at C-terminus in response to low pH. After purification of the Ei-PAL from rice seeds, intein cleavage was triggered by decreasing the buffer pH to release the target PAL protein from the Ei tag.

## Results

### Accumulation of ELP-intein-PAL fusion protein in rice seeds

To test the system, we constructed two expression vectors, SP and SA, for rice transformation (Figure 1): SP vector directed the expression of PAL while SA the ELP-intein-PAL (EiP) fusion protein. Driven by the seed-specific promoter of glutelin gene which encodes the major storage protein in rice, PAL and EiP would be expressed and accumulated in seeds. Mature T2 rice seeds harboring the SP and SA constructs were harvested and analyzed.

**Figure 2:**
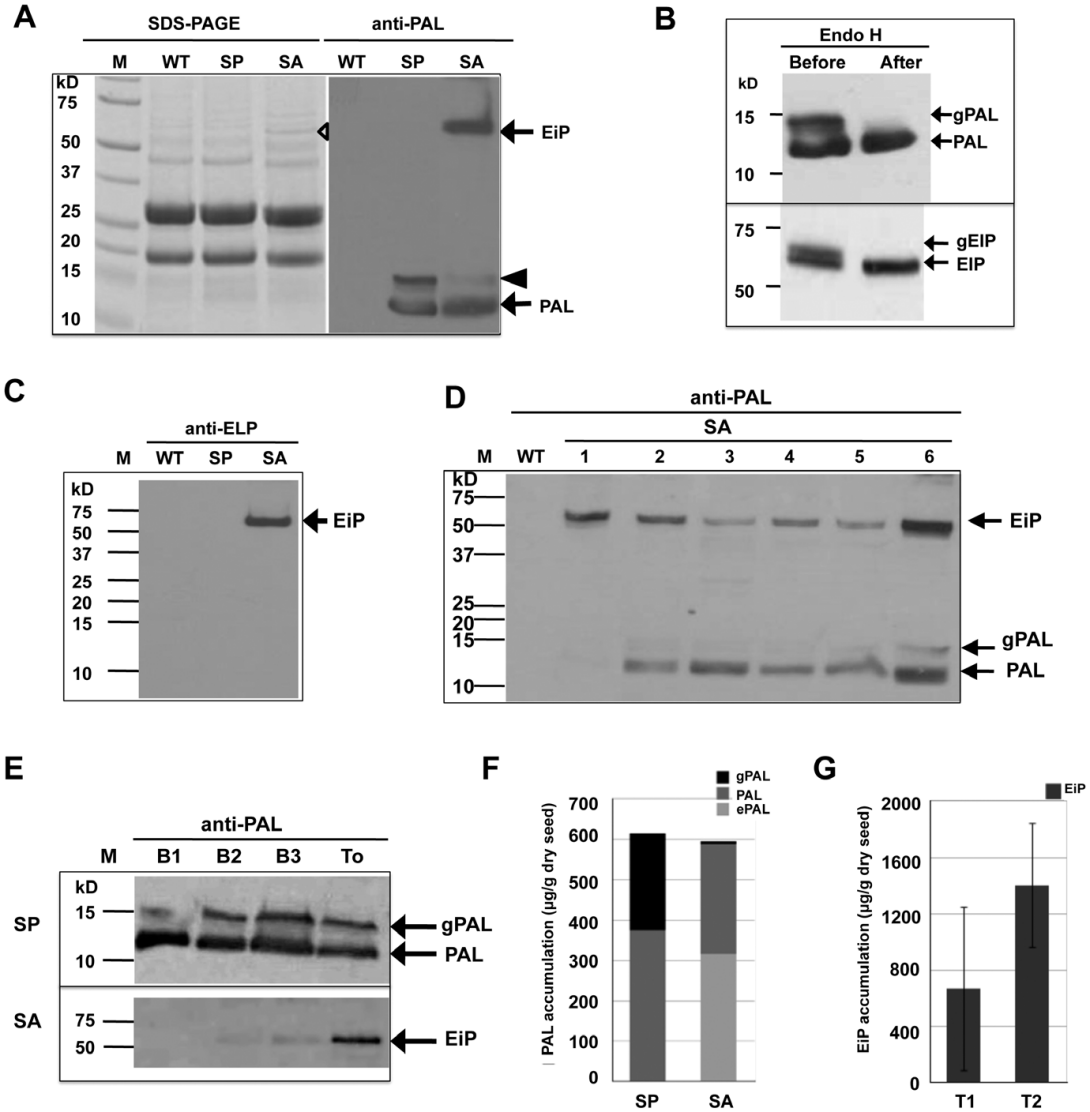
Expression of PAL in transgenic rice seeds. (A) Analysis of total protein extracted from transgenic rice seeds by SDS-PAGE and by immunoblot using anti-PAL antibody, anti-PAL. WT, non-trangenic rice; and M, Precision plus protein Standards (Bio-Rad). Empty trangle denoted the band of synthesized EiP in SA seeds and black triangle denoted the immunoactive band of N-glycosylated PAL. Arrows denoted PAL and ELP-intein-PAL fusion protein (EiP). (B) N-Glycosylation analysis of PAL in SP (top panel) and EiP in SA seeds (bottom panel) by Endo H digestion. gPAL, N-glycosylated PAL and gEiP, N-glycosylated EiP. (C) Detection of Ei tag in total protein extracted from SA seeds by immunoblot using anti-ELP antibody. Only EiP fusion protein was detected while free Ei tag was not observed. Arrows denoted N-glycosylated PAL (gPAL), PAL and ELP-intein-PAL fusion protein (EiP). (D) Immunoblot analysis on total protein extracted from SA seeds of different trnagenic lines (1–6) using anti-PAL antibody. (E) Immunoblot analysis of recombinant proteins extraction from transgenic rice seeds by four different extraction buffers, B1, B2, B3 and To. Total protein samples extracted from equal amount of rice seed powder by different buffers (see Materials and Methods) were used for analysis. (F) Relative accumulation levels of PAL in SP and SA rice seeds. gPAL, N-glycosylated PAL; and ePAL, relative amount of PAL derived from ELP-intein-PAL fusion protein. (G) Accumulation levels of uncleaved EiP in T1 and T2 seeds of SA transformed rice. Error bars indicate standard deviation among different transgenic lines.

As shown in Figure 2A, PAL protein was synthesized in SP rice seeds with expected molecular weight (MW) of 12.5 kD (refer to protein sequence in Figure S2A) while a protein band of slightly higher MW was also observed. After Endo H digestion for N-linked glycans removal, the upper band disappeared and the lower band became more intense in immunoblot analysis (Figure 2B, upper panel), indicating that a portion of PAL was modified by N-linked glycosylation. Endo H enzyme cleaves only N-linked glycan chains of high-mannose and hybrid type while it can not recognize the complex glycans processed by Golgi, suggesting that the recombinant PAL expressed in rice seeds didn't pass through Golgi.

In SA transformed rice seeds, compared with the banding patterns of WT and SP seeds, a newly emerged protein band with a MW around 56–60 kD, which is the expected size of EiP fusion protein, was observed in SDS-PAGE analysis (Figure 2A, empty triangle). Immunoblot analysis using anti-PAL antibody detected an immunoactive band at the same position (Figure 2A, right panel), suggesting that the identity of the newly emerged band was EiP fusion protein which was synthesized in transgenic rice seeds. Treatment with Endo H digestion led to the disappearance of the upper band, indicating that EiP was also partially glycosylated (Figure 2B, lower panel). Based on N-glycosylation prediction (Figure S2B), glycosylation will occur only on PAL, suggesting that ELP-intein fusion did not assert any adverse effect on the glycosylation of PAL. As in SP seeds, free PAL also appeared in SA seed samples (Figure 2A, D), suggesting that part of the EiP fusion protein was self-cleaved *in vivo*. However, when total protein extract was probed with anti-ELP antibody, only EiP fusion protein was detected while free Ei tag was not observed (Figure 2C), suggesting a possible occurance of degradation to the cleaved Ei tag.

The relative accumulation of PAL protein in the SP and SA seeds, on average, amounted to 615 µg and 595 µg/g dry seeds respectively (Figure 2F), suggesting that fusion with Ei tag did not affect the accumulation of PAL noticeably. When only the *in vivo* uncleaved EiP fusion protein was accounted for, the level of EiP protein accumulation in T2 seeds of SA transgenic rice, as determined by immunoblot analysis, amounted to 1.40 mg/g dry seeds on average (Figure 2G) and up to 2.94 mg maximum, representing significant amounts (2–4.2%) of the total seed protein.

### Subcellular localization of EiP protein

**Figure 3:**
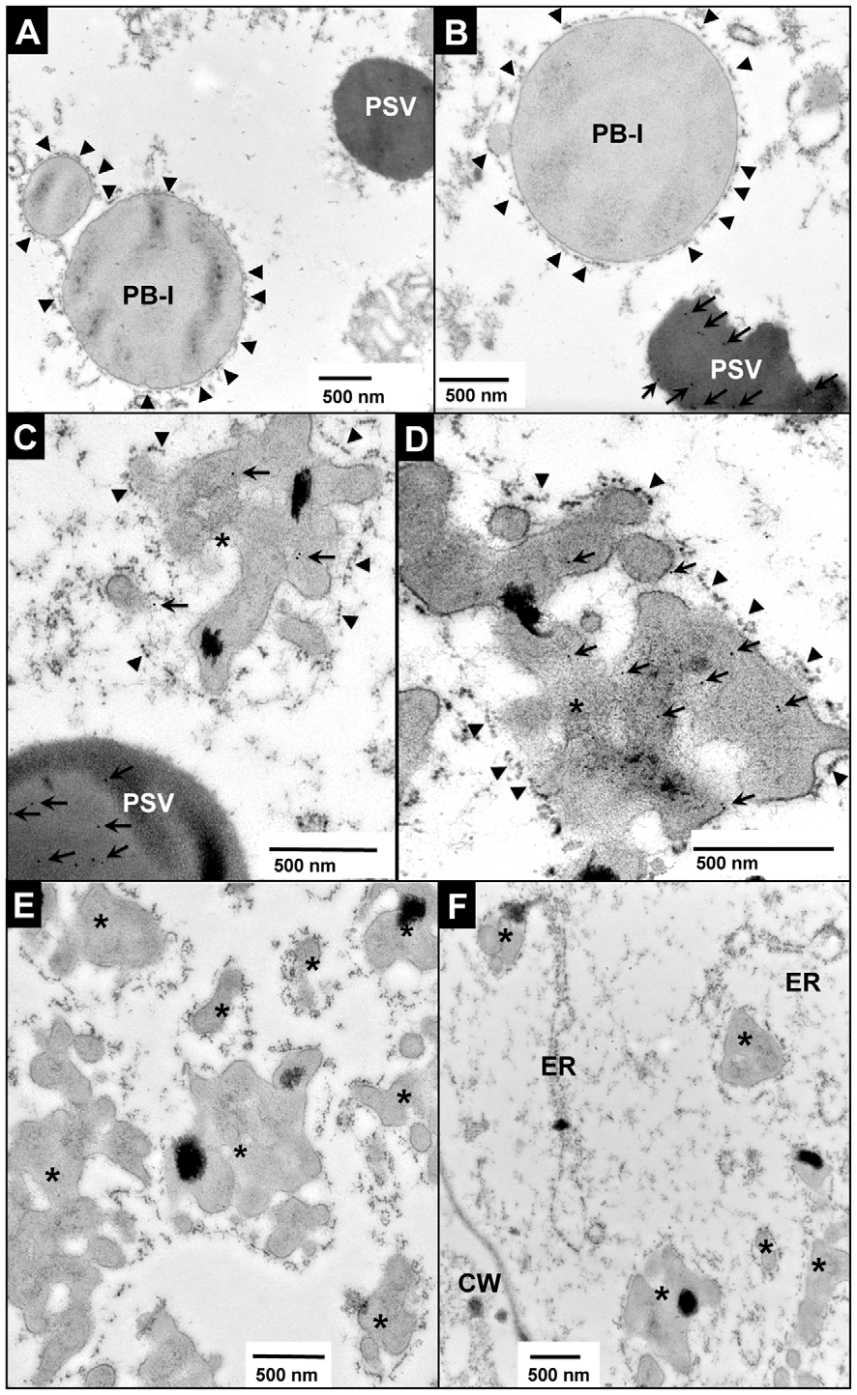
Subcellular localization of PAL in endosperm cells of transgenic rice seeds. (A–B) Immunogold labeling of WT (A) and SP (B) endosperm cells using anti-PAL antibody. Gold particles were indicated by arrows. (C–D) Immunogold labeling of SA endosperm cells using anti-PAL antibody (C) and with anti-ELP antibody (D). (E) Novel irregular protein bodies, iPBs, as indicated by *, were formed in endosperm cells of SA seeds. (F) The iPBs at the protein body-ER site were also observed. Arrows indicated the labeled gold particles while arrowheads ribosomes on rough ER membrane. PSV, protein storage vacuoles; CW, cell wall; PB-I, type I protein bodies; and bar, 500 nm.

In transgenic rice seeds, the expressed PAL could be easily extracted by soluble protein extraction buffer but not so as EiP fusion protein, which required the addition of SDS, urea and β-mercaptoethanol for complete extraction (Figure 2E). The insolubility of EiP fusion protein is likely due to its subcellular localization. The subcellular localization of recombinant proteins was examined by immuno-electron microscopy of immature transgenic rice seeds. As shown in Figure 3, PAL was deposited in protein storage vacuole (PSV), not in other subcellular organelles, such as type I protein body (PB-I) (Figure 3B), and no gold labeling was observed in PSV in WT endosperm cells (Figure 3A), confirming the positive labeling of SP samples. As glycosylation of PAL by Golgi was not observed (see above), PAL was likely transported into PSV bypassing Golgi. In the endosperm cells of SA rice seeds, anti-PAL antibody labeled PSV as well as novel irregular protein bodies (iPBs) (Figure 3C) while anti-ELP antibody only labeled iPBs in the endosperm cells of SA seeds (Figure 3D), suggesting that EiP fusion protein participated in the formation of these iPBs while the self-cleaved PAL was targeted into PSV. These EiP-containing iPBs varied in shape (0.3–3 µm in diameter) and appeared in clusters (Figure 3E). They were surrounded by ribosome-studded ER membrane (Figure 3C, D and E) and probably derived from ER, as iPBs in formation could be observed in ER (Figure 3F).

**Figure 4:**
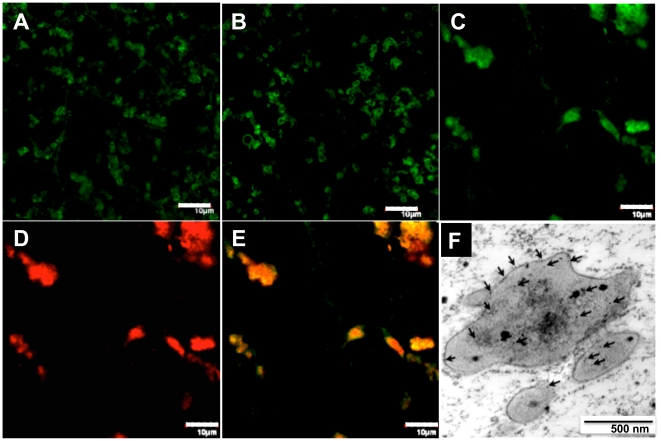
Co-localization of EiP and prolamins in the irregular protein bodies. (A–C) Prolamins labeling with FITC fluorescence in WT (A), SP (B) and SA (C) endosperm cells. (D) ELP labeling with Rhodamine Red fluorescence in SA endosperm cells. (E) Merged pictures of (C) and (D) Bars, 10 µm. (F) Immuno-gold labeling with anti-prolamin antibody confirmed the deposition of prolamins in the iPBs (indicated by arrows). Bar, 500 nm.

Prolamin, an abundant water-insoluble storage protein in rice seeds, is known to deposit in ER-derived type-I protein bodies (PB-I) [27], [28], while there were also several studies reported on the co-deposition of recombinant proteins with endogenous prolamins [29], [30]. In this study, through immuno-fluorescence microscopy analysis, multiple dispersed prolamin-deposited protein bodies were observed in WT and SP seeds (Figure 4A, B), while in SA seeds, prolamin-deposited protein bodies in irregular aggregates were observed (Figure 4C). Double labeling using anti-prolamin and anti-ELP antibodies showed that prolamins and ELP co-localizated in these aggregated structures (Figure 4C–E) and immuno-gold labeling with anti-prolamin antibody confirmed the deposition of prolamins in the iPBs (Figure 4F), suggesting that EiP might have participated in the formation of iPBs by retention of the water-insoluble prolamins, resulting in the difficulty encountered during extraction of EiP proteins from rice seeds.

### Purification of PAL protein from rice seeds by ELP-intein system

**Figure 5:**
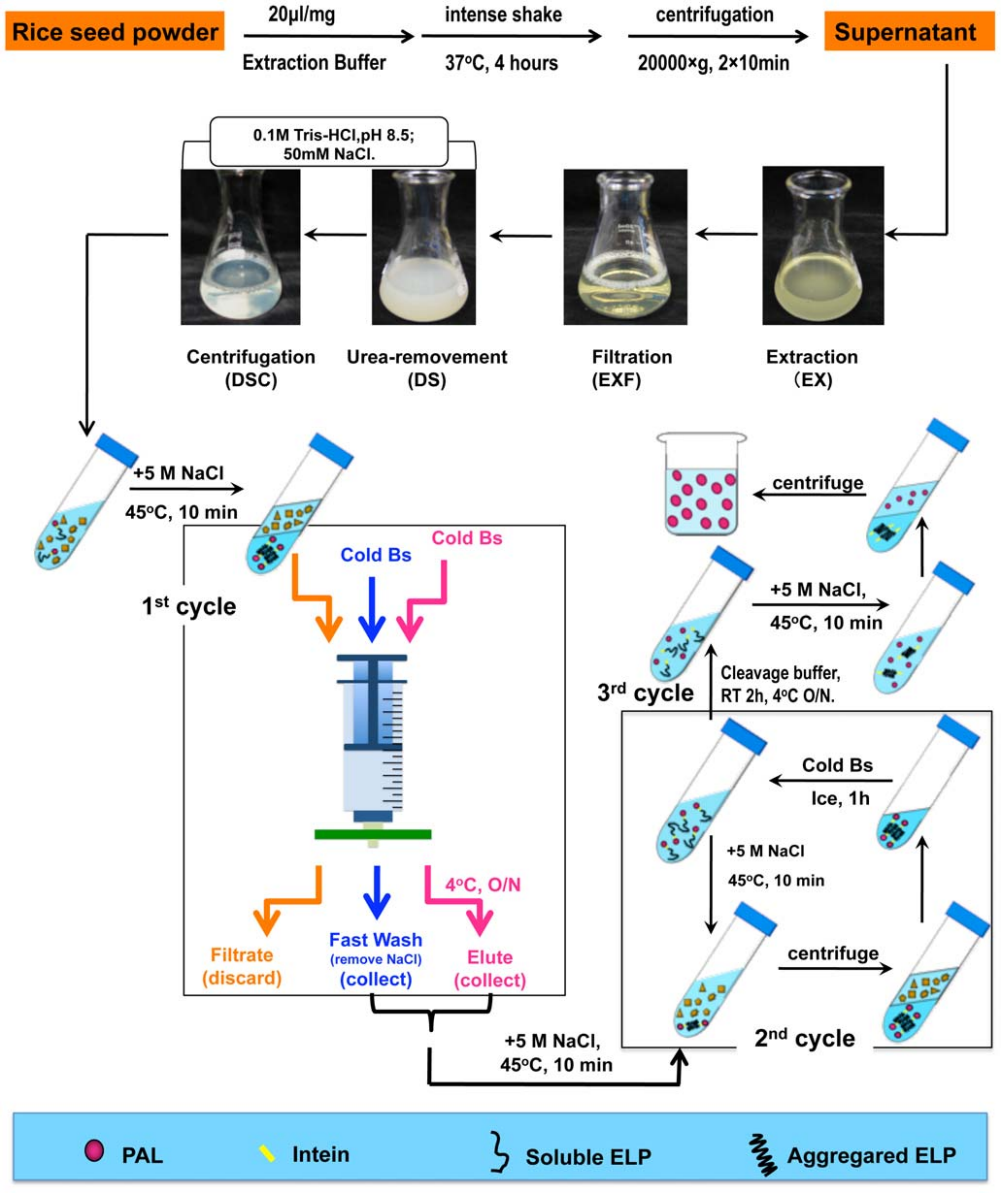
Schematic illustration of the purification of ELP-intein fusion protein from rice seeds.

Our main goal of this study is to demonstrate the feasibility of applying the ELP-intein system to plant bioreactor. Positive results were obtained in that efficient purification of the exemplary recombinant protein was achieved. The whole procedure starting from total protein extraction to final purification was highlighted and summarized schematically in Figure 5. In general, two to three cycles of phase transition of ELP are needed to obtain pure EiP protein, and about 2–3 days are required due to the involvement of overnight incubation in two steps.

**Figure 6:**
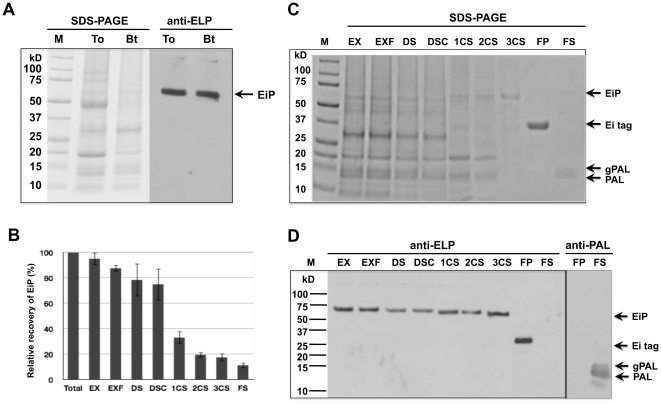
Purification of recombinant PAL from transgenic rice seeds by the ELP-intein system. The whole purification was targeted at EiP fusion protein without considering the *in vivo* cleaved PAL. (A) Analysis of total protein by SDS-PAGE and by immunoblot using anti-ELP antibody, anti-ELP. Equal amount of total protein was loaded in all lanes. To, extracted by total protein extraction buffer; Bt, extracted by Bt buffer. (B) Relative purification efficiency of EiP from transgenic rice seeds. Samples EX, EXF, DS, DSC, 1CS, 2CS, 3CS, FP and FS, as denoted in the Purification Scheme and Materials and Methods. Recovery percentage of EiP was counted relative to EiP extracted by total protein extraction buffer, and error bar was obtained from three independent experiments. (C–D) Purification of PAL from rice seeds by the ELP-intein system: (C) SDS-PAGE analysis; (D) immunoblot reacted with anti-ELP antibody, anti-ELP and anti-PAL antibody, anti-PAL. Lanes EX, EXF, DS, DSC, 1CS, 2CS, 3CS, FP and FS, as denoted in the Purification Scheme and Materials and Methods; and M, Precision Plus protein Standards (Bio-Rad). Arrows indicated ELP-intein-PAL fusion protein (EiP), cleaved ELP-intein tag (Ei), and purified PAL and N-glycosylated PAL (gPAL).

In the study, because SDS is known for difficulty in its complete removal from proteins after treatment, the extraction buffer was optimized by using mild detergent Tween-20 and high concentration of urea (6–8 M) to increase its solubilization and denaturing strength and to prevent disulfide bonds from formation. Before purification, several steps including filtration to remove debris, desalting to remove urea and centrifugation to remove insoluble compositions from the sample (Figure 5) were carried out to facilitate subsequent purification steps. As shown in Figure 6A and 6B, the optimized extraction buffer could extract 85–95% of the total EiP protein.

The inverse transition temperature (Tt) of ELP can be increased by reducing the length of the repeating pentapeptides or the concentration of ELP proteins, and decreased by increasing the length or the concentration of ELP, or by addition of salt [19]. In our purification system, ELP comprised of 60 repeating VPGXG was used to reduce the possibility of ELP phase transition from occurring *in vivo* due to possible high growth temperature, sometimes up to 40°C in summer time, encountered by the rice plants. As rather short length of ELP fusion tag was used in rice seeds, to trigger EiP aggregation during purification, high salt concentration and temperature are required, thus NaCl at 5 M and temperature at 40–45°C were used in this study.

We found that resolubilization of the aggregated target fusion protein in the 1^st^ cycle was always inefficient, possibly due to interference from the various cellular components in the total protein extract. Microfiltration method [31] was thus used with modifications in the 1^st^ cycle as described in Materials and Methods and shown in Figure 5. By gravity, the solution in syringe passed through the filter slowly, and by continuous flow of fresh buffer through large area of filter, it would facilitate the refolding of the aggregated EiP. For other cycles, normal centrifugation method was used. Purified EiP fusion protein could be observed in SDS-PAGE after 2 to 3 cycles (Figure 6C and 6D). At the cleavage step, pH shift achieved by adding Tris buffer at low pH initiated the cleavage reaction of intein, but longer incubation, such as 24 to 36 hours at 4°C, is recommended for complete cleavage, while freezing/thawing can be used as well to accelerate the cleavage. The cleavage and subsequent purification steps were efficient as no Ei tag remained in the final supernatant of PAL (Figure 6D).

Throughout the whole purification process, the greatest loss of EiP occurred at the resolubilization step in the 1^st^ cycle (Figure 6B). Although microfiltration resulted in positive effect on EiP resolubilization, only 30–40% EiP was recovered in this cycle. The loss of EiP in the other steps was relative low except that the refolding efficiency might affect EiP recovery in the desalting process. Through this highly optimized effort, the yield of pure PAL protein by the current method was up to 1.1 mg/g total seed protein. Rice seeds comprise 7–8% of protein (by dry weight), thus about 14 kg rice seeds can yield 1 g pure PAL protein.

## Discussion

In recent years, plant bioreactor has attracted increasing attention for its application to pharmaceutical protein production, but the low protein yield and high-cost downstream purification hamper its development. Much effort has been made to solve these problems, and recently, ELP fusion was shown to be a promising system in plant bioreactor with a potential of expression augment up to 40 folds and efficient recovery during purification [11], [23], [26], [32]. It was also found that C-terminal orientation of ELP fusion could produce higher level of target protein, relative to the N-terminal ELP fusion [26]. In our study, we inserted 60-repeat ELP encoded by preferred codons of rice to the N-terminus of target PAL protein, and didn't find increase in PAL accumulation, proving again that N-terminal orientation of ELP will not enhance protein yield. Therefore, C-terminal orientation of ELP is preferable for protein expression and purification in plant bioreactors and cleavage reaction at the N-terminus of intein should be targeted correspondingly. However, it should be noted that N-terminal cleavage reaction of intein requires additional chemicals, such as β-mercaptoethanol or DTT, to trigger and removal of these chemicals from the final products will incur additional cost in the purification.

Many pharmaceutical proteins are glycoproteins and the sugar moieties are essential for their stability and bioactivity, thus glycosylation should be a factor of consideration during production of proteins in transgenic plants. In our study, based on molecular weight estimation, it appeared that fusion of target protein to ELP-intein tag didn't affect its N-glycosylation. It is also known that glycosylation in plants differs from mammalian cells, especially glycosylation processed through Golgi apparatus. Many efforts were made to control the glycosylation of recombinant proteins in plants, such as knockin or knockout of related enzymes and retention of the target protein in ER [33]–[35]. In our case, fusion EiP was deposited in ER-derived protein body, thus no further modification would come from Golgi, which may provide an alternative way to produce recombinant proteins in transgenic plants.

In our study, partial *in vivo* self-cleavage of EiP was observed in transgenic rice. However, we did not observe such self-cleavage of EiP in *E.coli* cells (data not shown). Theoretically, intein cleavage should not happen largely under the natural pH of ER environment. Thus further research is needed to identify the underlying mechanism of such self-cleavage in rice. In this study, only the self-cleaved PAL protein was detected but not the free Ei tag. Endoplasmic Reticulum Associated Protein Degradation (ERAD) pathway is thought to direct ubiquitin-mediated degradation of ER-associated proteins [36]–[39]. Considering that the hydrophobic ELP polypeptide may be easily recognized as ERAD substrate and two possible ubiquitination sites were predicted within intein protein (http://www.ubpred.org/index.html) but not in PAL (data not shown), the cleaved Ei tag may have been degraded through the ERAD pathway.

Although *in vivo* self-cleavage is not desirable, the occurance of such cleavage in EiP in the present case may offer a new way to study protein quality control and protein sorting in plant ER. We observed that the *in vivo* self-cleavage didn't affect the transport of the cleaved PAL protein into PSV while the cleaved Ei tag was probably degraded, suggesting that self-cleavage might trigger the occurrence of some unclear sorting mechanisms. As protein quality control and protein sorting of ER in plants is still not clear, through analysis of the cellular events of *in vivo* self-cleavage of ELP-intein fusion protein, new information may be gained.

It has been reported that the hydrophobic nature of ELP might lead the KDEL retention signal containing ELP-GFP fusion protein to form ER-derived spherical protein bodies in transgenic tobacco leaves [21]. In our case, EiP may have aggregated with the hydrophobic prolamins in similar way and the aggregation in turn disturbs the normal formation of prolamin-deposited type I protein body (PB-I) and distorts the shape of protein bodies as reported previously [30]. However, as no ER retention signal was involved in this study, whether the retention of EiP proteins to ER was induced by ELP aggregation under high concentration of calcium within ER or by reaction with prolamins requires further research. On the other hand, the aggregation of EiP fusion protein with endogenous prolamins might have led to the poor solubility of EiP in this study, thus the application of EiP system in non-seed tissues, such as suspension cell or leaves, may avoid or reduce the undesirable aggregation, so as to achieve high efficiency in extraction and recovery of recombinant protein. However, compared with other tissues, plant seeds are the leading platform for recombinant protein production because of their several advantages such as high protein yields and stable storage, which contribute to the high expression level and low degradation of the target proteins [40]. Therefore, when non-seed tissues are involved, the productivity and stability of recombinant protein should be a factor of consideration.

Our study reported a successful application of the ELP-intein system to purify an exemplary recombinant protein from transgenic rice seeds, demonstrating that it is feasible to apply the ELP-intein system in plant bioreactors. Although EiP fusion protein was self-cleaved partially, the intact EiP fusion protein could still be efficiently purified from the rice seeds and pure PAL target protein can be obtained in significant amount. In this purification procedure, only centrifugation, filtration and manipulation of temperature, pH, and simple chemical conditions are required while no expensive chemicals and special instruments are involved. The whole procedure can be easily scaled up for industrial production, providing a cost-effective purification system for plant production platform.

PAL belongs to monocot mannose-binding lectins which have been shown to possess anti-virus and anti-tumor bioactivities due to their binding reaction with saccharide determinants or glycoconjugates present on tumor cell and virus surface [41]–[45]. We tested the bioactivity of PAL protein purified by the ELP-intein system from rice seeds using the proliferation inhibitory activity analysis on several different human cancer-cell lines. Results showed that the PAL purified by the ELP-intein system exhibited similar dose-dependent inhibition activity to PAL protein purified from *E.coli* (data not shown), indicating that the ELP-intein fusion expression and purification system did not influence the bioactivity of the recombinant protein.

In conclusion, this study investigated the application of ELP-intein fusion system for recombinant protein expression and purification in transgenic plants and demonstrated the successful purification of a recombinant protein retaining its bioactivity from transgenic rice seeds by the system. Although further studies are required to explore full molecular and cellular events on ELP-intein fusion expression in transgenic plants, such as the occurrence of *in vivo* self-cleavage, degradation of *in vivo* self-cleaved ELP-intein tag and formation of ER-derived protein bodies, the ELP-intein fusion system can undoubtedly offer an alternative way to achieve cost-effective downstream purification of recombinant proteins from plant production platform.

## Materials and Methods

### Ethics statement

All animal procedures involved in this study were approved by Animal Experimentation Ethics Committee, The Chinese University of Hong Kong (Committee Approval No. 08/051/MIS, and Animal License No. 08-157 in DH/HA&P/8/2/1 Pt13), and performed in accordance with all requirements of the relevant legislation of the Government of Hong Kong (HK), University by-laws and guiding principles and the HK Code of Practice: Care and Use of Animals for Experimental Purposes (the Code of Practice).

### Vector construction

The expression cassettes, SP and SA, for PAL and ELP-intein-PAL fusion protein expression, respectively, driven by rice glutelin *Glu*A (Gt1) promoter and signal peptide (Figure 1), were cloned into the multiple cloning sites of the T-DNA binary vector pSB130 [46].

The PAL gene, a monocot mannose-binding lectin gene, was cloned from *Pandanus amaryllifolius* by similar method as described by Chai *et al.* [47]. The ELP60 gene was generated from six repeats of the ELP10 gene (Figure S1) by similar methods as described by Scheller *et al.* [48]. Ssp DnaB intein and linker gene were obtained from pTWIN2 vector (5902–5940 bp for linker gene; 5941–6402 bp for intein gene) purchased from NEB (http://www.neb.com/nebecomm/products/productN6952.asp). Both ELP10 and intein genes were optimized for rice preferred codons and synthesized by GenScript Corporation.

### Agrobacterium-mediated transformation

The chimeric genes in pSB 130 expression vectors were transformed into *Agrobacterium tumefaciens* EHA105 by electroporation. After two weeks inducation, calli of *japonica* cv. 9983 were used for rice transformation. *Agrobacterium*-mediated transformation, selection and regeneration were performed following the protocol provided by CAMBIA (http://www.cambia.org/daisy/cambia/4214.html). Regenerated transgenic rice plantlets were transferred to soil and grown in facilities for transgenic plants at The Chinese University of Hong Kong. Positive transgenic rice plants were determined by PCR screening and Southern blot analysis. Mature positive transgenic rice seeds were collected as T1 seeds. T2 seeds generated from positive T1 plants were used for protein expression analysis and purification experiments.

### Protein extraction and immunoblot analysis

Mature rice seeds were ground into powder by a blender. Total protein extraction buffer (To buffer; 0.1 M Tris-HCl, pH 8.5, 50 mM NaCl, 5% SDS, 4 M urea, 5% β-mecaptoethanol) was added to the seed powder with a ratio of 20 µl/mg, and incubated at 35–37°C with intense shaking for 2–4 hours. The whole homogenate was centrifuged at 20,000×g for 10 min at room temperature twice. Supernatant was collected as total seed protein for expression analysis. B1 buffer [0.1 M Tris-HCl (pH 8.5), 50 mM NaCl], B2 buffer [0.1 M Tris-HCl (pH 8.5), 50 mM NaCl, 0.5% SDS] and B3 buffer [0.1 M Tris-HCl (pH 8.5), 50 mM NaCl, 0.5% SDS, 4 M urea] were used to test extraction efficiency of recombinant proteins.

For immunoblot analysis, total seed protein in loading buffer (50 mM Tris-HCl, pH 8.5, 2% SDS; 10% Glycerol;1% β-mecaptoethanol; 0.02% Bromophenol Blue) was separated by 15% SDS-PAGE and transferred to PVDF membrane. Immunoblot analysis was carried out with anti-PAL primary antibody from rabbit and anti-Rabbit IgG–Peroxidase antibody (Sigma). Recombinant PAL and ELP produced by *E.coli* (see Antibody Preparation as below) were used as positive controls. To estimate the expression levels of PAL and EiP fusion protein in transgenic rice seeds, the same amount of total seed protein from different transgenic lines was loaded onto SDS-PAGE during immunoblotting while leaving 3 lanes for positive control standards of PAL or ELP at 20, 50 and 100 ng/lane, respectively. The detected immunoactive bands of the experiemtntal samples were compared with the positive controls and quantified by densitometry using the ImageJ software (National Institute of Health, USA; http://rsbweb.nih.gov/ij/). All the quantity of samples fell within the three control standard amount.

### N-linked glycosylation analysis

Total protein samples (10 µg) extracted by B1 buffer from SP rice seeds and by B2 buffer from SA rice seeds were denatured by Glycoprotein Denaturing Buffer (New England Biolabs) for 10 min at 100°C and then incubated with 2 mU of endoglycosidase H (Endo H, New England Biolabs) in the buffer provided for 1 h at 37°C. After digestion, samples were analyzed by immunoblotting with anti-PAL antibody in the presence of a negative control consisting of equal amount of total protein without the enzyme treatment.

### Purification by ELP-intein system

Total protein was extracted by Bt buffer (0.1 M Tris-Cl, pH 8.5; 50 mM NaCl; 6 M Urea; 0.5% Tween-20). The protein extract (EX) was filtered to remove debris (EXF); desalted by PD-10 column (GE Healthcare) or dialysis with Bp Buffer (0.1 M Tris-HCl, pH 8.5; 50 mM NaCl) to remove urea (DS); and then centrifuged at 20,000×g for 10 min at 4°C to remove any insoluble debris (DSC). The supernatant was collected for further purification.

To trigger the temperature-sensitive inverse phase transition of ELP, 5 M NaCl was added to the samples at a ratio of 1∶1 (v/v) and the mixture was incubated at 45°C for 10 min. The 1^st^ purification cycle was performed as described by Ge *et al*. [31] with some modifications. The NaCl-treated sample was transferred to a syringe equipped with a Millipore filter (PES membrane, 0.22 µm) and the filtrate was discarded while the aggregated ELP-intein-PAL fusion protein remained on the filter. One ml cold Bs buffer (0.1 M Tris-HCl, pH 8.5; 50 mM NaCl; 0.1% Tween-20) was passed through quickly to remove additional NaCl from the filter and the wash was collected as some fusion protein might return to soluble state and pass the filter. Another 1–3 ml (or 1/10 volume of original TEP sample) cold Bs buffer was added into the syringe without a plunger, and the whole system was kept at 4°C overnight (O/N) with a tube below the filter to collect the elute (soluble EiP) by gravity. Any remaining solution (soluble EiP) in the syringe was collected by pushing with a plunger. The elution and wash parts were combined as sample 1CS.

The 2^nd^ cycle was carried out by the inverse phase transition procedure as reported by Wu *et al*. [20]. NaCl (5 M) was added at a ratio of 1∶1 v/v and the mixture was incubated at 45°C for 10 min, followed by immediate centrifugation. The pellet was resuspended in cold Bs buffer with 1/5–1/2 original volume and kept on ice for 1 hour with gentle agitation. After centrifugation at 4°C, the supernatant was collected as 2CS sample. The 3^rd^ cycle was performed as the 2^nd^ cycle except that 50 mM PBS buffer (pH 7.2) was used to resolubilize the pellet. The supernatant was collected as 3CS sample.

To trigger the cleavage reaction by intein, 1/10 volume of 1 M Tris-HCl (pH 4.5) was added to the 3CS sample to a final pH value at 6.0–6.5. The sample was allowed to cleave at room temperature for 2 hours and then 4°C overnight (for complete cleavage). Another phase transition was performed by addition of NaCl followed by centrifugation. The supernatant was collected and desalted as final purified target protein (FS) and the pellet was resuspended as final ELP-intein tag (FP).

### Antibody preparation

Complementary DNAs for ELP and PAL were cloned into pET-30a (Novagen) and expressed in *E. coli* (strain BL21 DE3, Novagen) as His-Tag fusion proteins. Both of the proteins were purified by Ni-NTA agrose (Qiagen) and injected into rabbits 4 times at 2-week intervals at the Laboratory Animal Services Center (LASC), The Chinese University of Hong Kong, Hong Kong. The final rabbit serum was collected and antibody titre was determined by dot blot analysis.

Polyclonal rabbit anti-prolamin antibody were kind gift from Ms. Kaman Ho of The Chinese University of Hong Kong.

### Immunofluorescence microscopy

Immature rice seeds (10–15 days after flowering) were fixed in FAA solution (50% ethanol, 10% formaldehyde, 5% acetic acid) at 4°C for 24 hours, dehydrated with automated Enclosed Tissue Processor (Leika ASP200 S) and embedded with paraffin wax. The embedded samples were sectioned (7 µm) on a rotary microtome and placed onto slides. After depapraffin, the sections were blocked in 5% BSA with PBS at room temperature for 2 hours (RT 2 hours) before labeling. Fab rhodamine-conjugated anti-rabbit IgG and FITC-conjugated anti-rabbit IgG were purchased from Jackson ImmunoResearch Laboratory (http://www.jacksonimmuno.com/). For double-labeling, refer to the online guidelines (http://www.jacksonimmuno.com/technical/techmain.asp). Fluorescence images were obtained by the Olympus FV1000 system with the FV10-ASW imaging software (Olympus).

### Immuno-gold electron microscopy (EM)

Immature seeds were collected at 10–12 days after flowering and fixed at 4°C for overnight in 4% (v/v) paraformaldehyde and 0.1% (v/v) glutaraldehyde buffered at pH 7.2 with 0.1 M PBS buffer. The fixed samples were dehydrated and embedded in LR White resin. Immunogold electron microscopy (EM) on ultrathin sections was performed using standard procedure with primary antibodies at 1∶50 dilution, and gold-coupled secondary antibodies at 1∶50. After post-staining by aqueous uranyl acetate/lead citrate, the ultrasections were examined in Hitachi H-7650 transmission EM with a CCD camera (Hitachi High-Tech, http://www.hitachi-hitec.com) operating at 80 kV.

## Supporting Information

Figure S1 **Gene and amino acid sequences of ELP for use in transgenic rice.** Main sequences of ELP10 gene and peptides were underlined. Lower-case letters represent restriction endonuclease sites for further sub-cloning. Bold italic letters represent the restriction endonuclease sites of PflMI and BglI in ELP60 synthesized from ELP10.
(TIF)

Figure S2 **PAL protein sequence and prediction of N-linked glycosylation sites.** (A) PAL protein sequence. Possible N-linked glycosylation sites were underlined. (B) N-linked glycosylation site prediction in EiP (http://www.cbs.dtu.dk/services/NetNGlyc/). Online explanation indicates that a position with a potential (vertical lines) of crossing the threshold (horizontal line at 0.5) is predicted as glycosylated. Two possible glycosylation sites in PAL were predicted, NKTR and NATL, while no possible site in ELP or intein fusion tag was predicted.
(TIF)
