# Supplementary figures and images for: A Cost-Effective ELP-Intein Coupling System for Recombinant Protein Purification from Plant Production Platform
Source: PLoS One. 2011 Aug 30;6(8):e24183. doi: 10.1371/journal.pone.0024183 (PMC3168869; doi:10.1371/journal.pone.0024183)

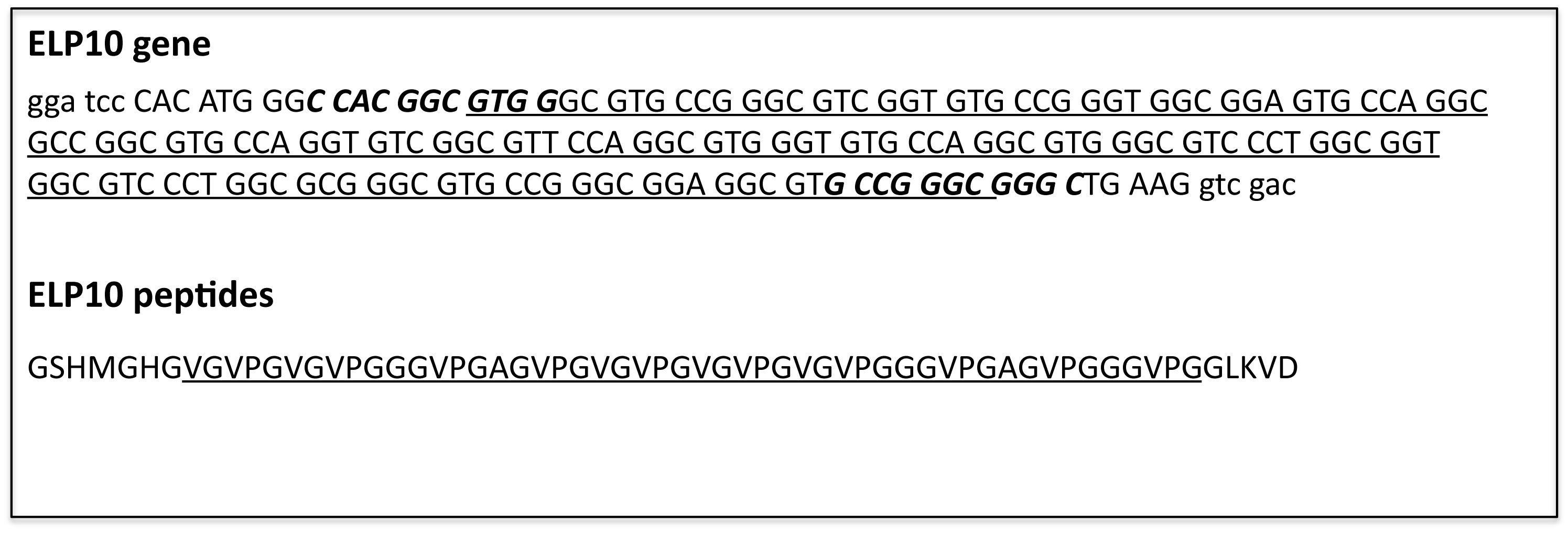

Supplement: Figure S1 — Gene and amino acid sequences of ELP for use in transgenic rice. Main sequences of ELP10 gene and peptides were underlined. Lower-case letters represent restriction endonuclease sites for further sub-cloning. Bold italic letters represent the restriction endonuclease sites of PflMI and BglI in ELP60 synthesized from ELP10. (TIF) [file pone.0024183.s001.tif]

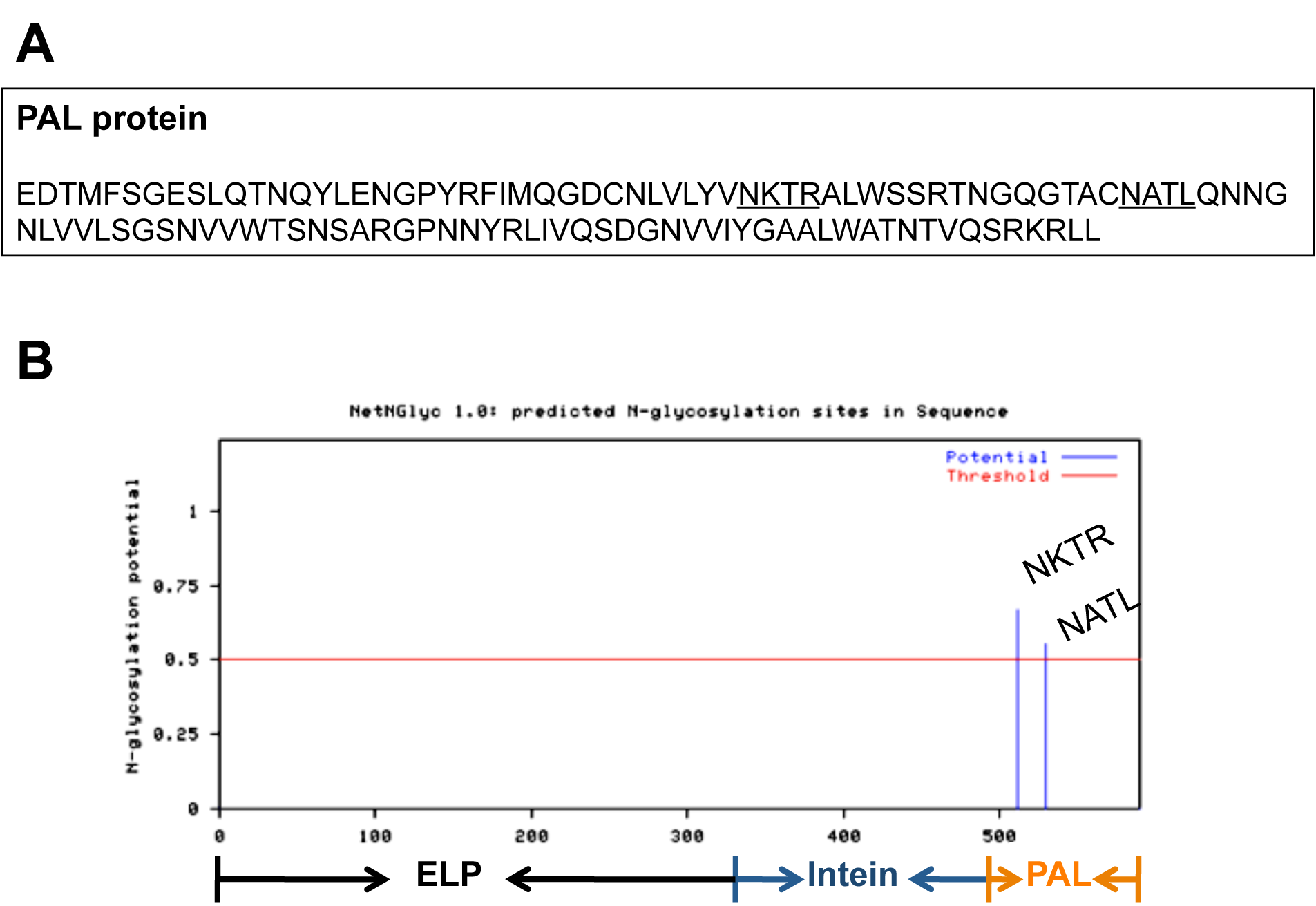

Supplement: Figure S2 — PAL protein sequence and prediction of N-linked glycosylation sites. (A) PAL protein sequence. Possible N-linked glycosylation sites were underlined. (B) N-linked glycosylation site prediction in EiP (http://www.cbs.dtu.dk/services/NetNGlyc/). Online explanation indicates that a position with a potential (vertical lines) of crossing the threshold (horizontal line at 0.5) is predicted as glycosylated. Two possible glycosylation sites in PAL were predicted, NKTR and NATL, while no possible site in ELP or intein fusion tag was predicted. (TIF) [file pone.0024183.s002.tif]
